# Structural insights of a highly potent pan-neutralizing SARS-CoV-2 human monoclonal antibody

**DOI:** 10.1073/pnas.2120976119

**Published:** 2022-05-12

**Authors:** Jonathan L. Torres, Gabriel Ozorowski, Emanuele Andreano, Hejun Liu, Jeffrey Copps, Giulia Piccini, Lorena Donnici, Matteo Conti, Cyril Planchais, Delphine Planas, Noemi Manganaro, Elisa Pantano, Ida Paciello, Piero Pileri, Timothée Bruel, Emanuele Montomoli, Hugo Mouquet, Olivier Schwartz, Claudia Sala, Raffaele De Francesco, Ian A. Wilson, Rino Rappuoli, Andrew B. Ward

**Affiliations:** ^a^Department of Integrative Structural and Computational Biology, The Scripps Research Institute, La Jolla, CA 92037;; ^b^Monoclonal Antibody Discovery (MAD) Lab, Fondazione Toscana Life Sciences, 53100 Siena, Italy;; ^c^VisMederi S.r.l, 53100 Siena, Italy;; ^d^Istituto Nazionale Genetica Molecolare INGM “Romeo ed Enrica Invernizzi”, 20122 Milan, Italy;; ^e^Laboratory of Humoral Immunology, Department of Immunology, Institut Pasteur, INSERM U1222, 75015 Paris, France;; ^f^Virus and Immunity Unit, Department of Virology, Institut Pasteur, CNRS UMR 3569, 75015 Paris, France;; ^g^Vaccine Research Institute, 94000 Creteil, France;; ^h^VisMederi Research S.r.l., 53100 Siena, Italy;; ^i^Department of Molecular and Developmental Medicine, University of Siena, 53100 Siena, Italy;; ^j^Department of Pharmacological and Biomolecular Sciences DiSFeB, University of Milan, 20122 Milan, Italy;; ^k^The Skaggs Institute for Chemical Biology, The Scripps Research Institute, La Jolla, CA 92037;; ^l^Department of Biotechnology, Chemistry and Pharmacy, University of Siena, 53100 Siena, Italy

**Keywords:** SARS-CoV-2, monoclonal therapy, neutralizing antibody, cryoelectron microscopy, variants of concern

## Abstract

As the coronavirus disease 2019 (COVID-19) pandemic continues, there is a strong need for highly potent monoclonal antibodies (mAbs) that are resistant against severe acute respiratory syndrome coronavirus 2 (SARS-CoV-2) variants of concern (VoCs). Here, we evaluate the potency of the previously described mAb J08 against these variants using cell-based assays and delve into the molecular details of the binding interaction using cryoelectron microscopy (cryo-EM) and X-ray crystallography. We show that mAb J08 has low nanomolar affinity against most VoCs and binds high on the receptor binding domain (RBD) ridge, away from many VoC mutations. These findings further validate the phase II/III human clinical trial underway using mAb J08 as a monoclonal therapy.

## Significance

Clinical candidate monoclonal antibody J08 binds the severe acute respiratory syndrome coronavirus 2 (SARS-CoV-2) S-protein independent of known escape mutations and is able to potently neutralize most variants of concern (VoCs). Here, we explore these properties using cell-based assays and structural studies. A relatively small epitope footprint high on the receptor binding domain (RBD) ridge and the ability to bind multiple conformational states of the S-protein contribute to strong neutralization across several variants.

To date, over 219 million cases and 4.5 million deaths worldwide have been caused by severe acute respiratory syndrome coronavirus 2 (SARS-CoV-2) (1), along with high levels of unemployment and far-reaching supply chain issues in the world’s economy. While the situation is vastly improving with numerous vaccine rollouts and over 6.03 billion doses of vaccines administered worldwide, they have been heavily skewed toward developed nations (2). Hence, disadvantaged communities and low- and middle-income countries remain potential hotspots for the emergence of viral variants with increased infectivity and mortality. Designated variants of concern (VoCs) pose the greatest threat to progress made thus far because data have shown that they can reduce the effectiveness of the coronavirus disease 2019 (COVID-19) vaccines (3). These findings have led vaccine manufacturers to test booster shots (4–6) that will ultimately result in another logistical distribution and administration challenge.

COVID-19 vaccines, whether mRNA, protein, or viral vector based, aim to provide acquired immunity and protection against serious disease by presenting the SARS-CoV-2 spike protein (S-protein) to the immune system (7). The S-protein is a glycosylated, homotrimeric type I transmembrane fusion protein responsible for host cell attachment via its receptor binding domain (RBD) to human angiotensin-converting enzyme 2 (hACE2), a process that is enhanced by a coreceptor, cellular heparan sulfate (8). The S-protein is made up of the S1 domain (residues 1 to 685) containing the RBD and N-terminal domain (NTD) and the S2 domain (residues 686 to 1213) housing the fusion machinery. Both the RBD and NTD are immunodominant epitopes that are targeted by a majority of neutralizing antibodies (9).

Ending the pandemic is being challenged by the emergence of VoCs, uneven roll out of vaccines worldwide, vaccine hesitancy, uncertainty of whether COVID-19 vaccines can prevent transmission, breakthrough infections among vaccinated populations, and if vaccinated and naturally infected individuals will have long-lasting immunity against the virus. Therefore, having readily available, highly potent, and viral-variant-resistant monoclonal antibodies (mAbs) may serve to mitigate the propagation of troublesome variants worldwide and treat those that remain vulnerable after vaccination. Early efforts with monoclonal therapies have had mixed results. LY-CoV555 (bamlanivimab), developed by Eli Lilly and Company, was found to have no significant effect on viral load compared to a placebo in phase II trials and is easily susceptible to escape mutations present in common VOCs (10, 11). Monoclonal mixtures are one strategy to decrease the chance of viral escape, and a recent Emergency Use Authorization was given for Regeneron’s casirivimab (REGN10933) and imdevimab (REGN10987) and for Eli Lilly and Company’s bamlanivimab and etesevimab combinations (10, 12).

In this study, we characterized the neutralization breadth of a highly potent human mAb, J08, previously isolated from a convalescent COVID-19 patient (13), against the SARS-CoV-2 B.1.1.7 (Alpha), B.1.351 (Beta), P.1 (Gamma), B.1.617.2 (Delta), and recently emerged B.1.1.529 (Omicron) VoCs. Following functional characterization, we further investigated the structural details of J08 to define the epitope that retains neutralization activity against these VoCs.

## Results

### J08 Cross-Neutralizes All Current SARS-CoV-2 VoCs.

**Fig. 1.**
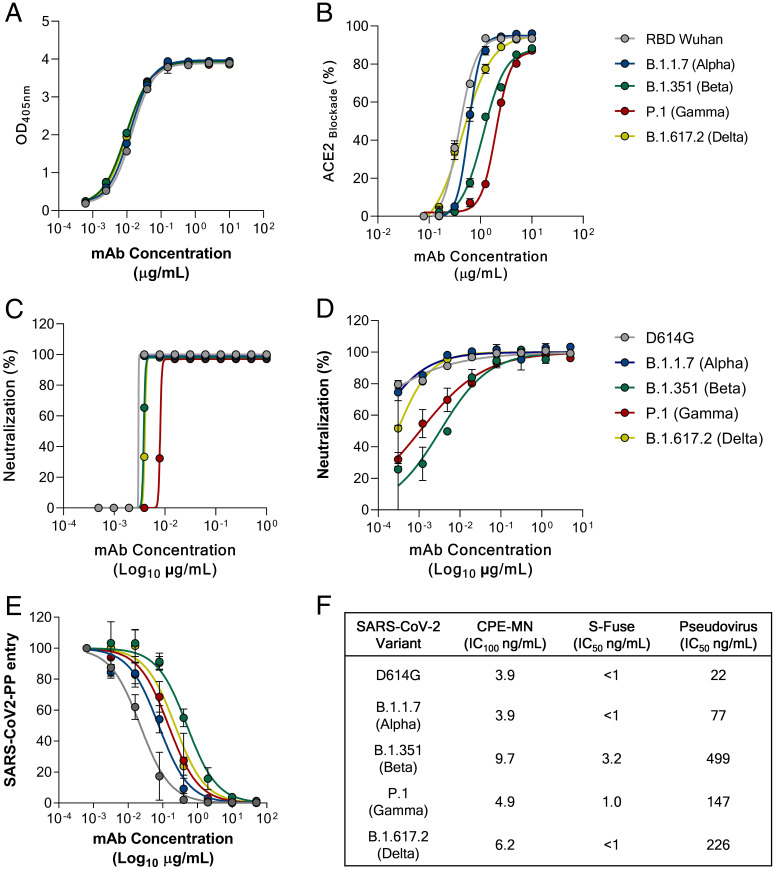
J08 activity against SARS-CoV-2 and emerging variants. Graphs show the ability of mAb J08 to bind (*A*); block RBD/ACE2 interaction (*B*); and neutralize SARS-CoV-2 D614G, B.1.1.7, B.1.351, P.1, and B.1.617.2 using a CPE-MN (*C*), S-fuse (*D*), and pseudovirus platform (*E*). (*F*) Summary of the IC_100_ and IC_50_ results obtained for all neutralization assays. Errors bars display SEM for *N* = 3 measurements.

To evaluate neutralization breadth, J08 was tested for binding, ACE2 blocking, and neutralization against the SARS-CoV-2 D614G virus and VoCs B.1.1.7 (isolated in the United Kingdom) (14), B.1.351 (isolated in South Africa) (15), P.1 (isolated in Brazil) (16), and B.1.617.2 (isolated in India) (17). These VoCs have been renamed by the World Health Organization as Alpha, Beta, Gamma, and Delta variants, respectively (18). We first tested antibody binding or ACE2 blocking in an enzyme-linked immunosorbent assay (ELISA) using the S-protein RBD from the original Wuhan strain and from the Alpha through Delta VoCs. J08 was able to bind and interfere with the RBD/ACE2 interaction with all tested variants (Fig. 1 *A* and *B*). We next evaluated the neutralization activity of J08 against authentic SARS-CoV-2 and VoC viruses using a cytopathic-effect-based microneutralization (CPE-MN) assay, an S-fuse neutralization assay, and a SARS-CoV-2 pseudovirus platform. The neutralization experiments were performed in three different and independent laboratories. The CPE-MN assay demonstrated that J08 was able to neutralize the SARS-CoV-2 D614G virus with a 100% inhibitory concentration (IC_100_) of 3.9 ng/mL and maintain its extremely high neutralization activity against all tested VoCs with an IC_100_ of 3.9, 9.7, 4.9, and 6.2 ng/mL for the Alpha, Beta, Gamma, and Delta VoCs, respectively (Fig. 1 *C* and *F*). A similar scenario was observed with the S-fusion neutralization assay, where J08 was able to neutralize all VoCs tested. The S-fusion neutralization assay differs from the other methods as it uses cell lines (U2OS-ACE2 GFP1-10 or GFP 11) that emit fluorescence when they are productively infected by SARS-CoV-2. The antibody neutralization activity is evaluated as the ability of the mAb to block infection and therefore syncytial formation and fluorescence emission. Given the extremely high neutralization potency of J08, we were not able to define a 50% inhibitory concentration (IC_50_) against the D614G, Alpha, and Delta variants, and therefore, we assigned it as <1 ng/mL. On the other hand, it was possible to define the neutralization potency of J08 against the Beta and Gamma variants that showed an IC_50_ of around 3.2 and 1.0 ng/mL, respectively (Fig. 1 *D* and *F*). Finally, we evaluated the neutralization activity of J08 using a lentiviral pseudovirus platform produced with a SARS-CoV-2 spike variant, which included a cytoplasmic tail deletion of 19 amino acids. Similar cytoplasmic tail deletions showed an enhanced spike incorporation into pseudovirions and increased viral entry into cells, compared to those with full-length S-protein (19). With our pseudovirus platform, we observed an overall lower neutralization IC_50_ by J08. The differences in measured inhibitory concentrations between assay types could arise from the pseudovirus platform used and its high incorporation of S-protein into pseudovirions, as well as the use of cell lines showing different levels of cell surface hACE2 presentation. In addition, the variations of neutralization potencies observed between authentic virus and pseudovirus might also be due to the diluted viral stocks used for these assays. Nonetheless, J08 showed an IC_50_ against the D614G, Alpha, Beta, Gamma, and Delta variants of 22, 77, 499, 147, and 226 ng/mL, respectively, further confirming its ability to neutralize all VoCs (Fig. 1 *E* and *F*). While we performed this work, the B.1.1.529 (Omicron) emerged and spread worldwide. Therefore, we evaluated the ability of J08 to neutralize this SARS-CoV-2 VoC. Despite that an important IC_100_ and IC_50_ reduction was observed in our CPE-MN and pseudovirus platform, J08 retained some neutralization activity against Omicron on the contrary of several mAbs recognizing a similar epitope region on the SARS-CoV-2 S-protein (20, 21) (*SI Appendix*, Fig. S1). Using biolayer interferometry, we detected appreciable affinity of J08 immunoglobulin G (IgG) to the Omicron-CoV-2-6P S-protein (dissociation constant [K_D_], ∼98 nM), but compared to the SARS-CoV-2-6P D614G S-protein, against which we did not observe a measurable off-rate under similar assay conditions, J08 dissociates relatively rapidly from the Omicron-CoV-2-6P S-protein (*SI Appendix*, Fig. S1 *D* and *E*). The on-rate is also ∼sevenfold slower against Omicron, suggesting that altered binding kinetics are decreasing the neutralization potency of J08 against this variant (*SI Appendix*, Fig. S1).

### Neutralization Activity of Competitor Antibodies against SARS-CoV-2 and Emerging Variants.

To compare the neutralization potency and breadth of J08 to other SARS-CoV-2 neutralizing mAbs that have received emergency use authorization for COVID-19 treatment, we recombinantly expressed REGN10987 (12), REGN10933 (12), S309 (22), CoV2.2196 (23), and LY-CoV016 (10) as immunoglobulin G1 (IgG1). All mAbs were tested by the CPE-MN assay using authentic SARS-CoV-2 viruses and by our pseudovirus platform against the D614G, Alpha, Beta, Gamma, and Delta VoCs. REGN10987 showed a neutralization potency of 24.6, 19.5, 4.9, 3.1, and 19.7 ng/mL against D614G, Alpha, Beta, Gamma, and Delta VoCs, respectively (*SI Appendix*, Fig. S2 *A* and *K*). A similar trend for these variants was observed with our pseudovirus platform, although the Delta variant showed a 52-fold reduction (*SI Appendix*, Fig. S2 *F* and *K*). Antibody REGN10933, which recognizes the receptor binding motif of the S-protein, showed high neutralization potency against D614G, Alpha, and Delta VoCs but was heavily impacted by the Beta and Gamma VoCs, showing a 59- and 29.5-fold decrease, respectively (*SI Appendix*, Fig. S2 *B* and *K*). Our pseudovirus platform results were in accordance with the CPE-MN assay (*SI Appendix*, Fig. S2 *G* and *K*). We then evaluated antibody S309, which targets a region outside of the S-protein receptor binding motif (22). This antibody, when assessed by the CPE-MN assay, retained its neutralization potency against all SARS-CoV-2 variants showing an IC_100_ of 156, 248, 78, 25, and 79 ng/mL for the D614G, Alpha, Beta, Gamma, and Delta VoCs, respectively (*SI Appendix*, Fig. S2 *C* and *K*). In our pseudovirus platform, S309 was able to neutralize all SARS-CoV-2 variants but in the 4- to 41-µg/mL range (*SI Appendix*, Fig. S2 *H* and *K*). Antibody CoV2-2196 showed high neutralization potency against all variants in both the CPE-MN assay and pseudovirus platform ranging from 12.3 to 49.2 ng/mL and 45.0 to 425.0 ng/mL, respectively (*SI Appendix*, Fig. S2 *D*, *I*, and *K*). Despite CoV2-2196 being the only antibody that showed high neutralization potency against all variants, J08 remains the only antibody able to neutralize all VoCs with an IC_100_ below 10 ng/mL Finally, we evaluated antibody LY-CoV016 that was heavily impacted by the Alpha variant with a 12-fold decrease in neutralization and was unable to bind to the Beta and Gamma variants (*SI Appendix*, Fig. S2 *E* and *K*). LY-CoV016 lost activity to Alpha, Beta, and Gamma but was not impacted by the Delta VoC, showing an IC_100_ of 49.6 ng/mL (*SI Appendix*, Fig. S2 *E* and *K*). When LY-CoV016 was tested in our pseudovirus platform, it was able to neutralize the D614G and Delta variants with a similar potency of around 300 ng/mL (*SI Appendix*, Fig. S2 *J* and *K*). In addition to previous SARS-CoV-2 VoCs, REGN10987, REGN10933, S309, CoV2-2196, and LY-CoV016 were tested against the recently emerged Omicron in our CPE-MN and pseudovirus platform. REGN and LY-CoV016 antibodies did not show neutralization activity against this VoC. Conversely, S309 and CoV2-2196 retained activity, albeit showing up to 3.2- and 1,600-fold reduction, respectively, in our CPE-MN assay (*SI Appendix*, Fig. S1). These results are consistent with previously reported neutralization activity of each antibody (11, 21, 24–30).

### J08 Can Bind Dynamic RBD Conformations.

**Fig. 2.**
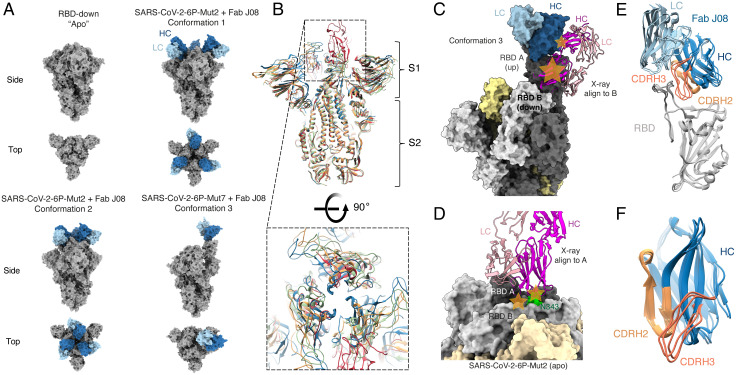
Conformational states of the S-protein-Fab J08 complex observed by cryo-EM. (*A*) *Top* and *Side* view surface representations of SARS-CoV-2-6P + Mut2, SARS-CoV-2-6P-Mut2 + Fab J08 conformation 1, SARS-CoV-2-6P-Mut2 + Fab J08 conformation 2, and SARS-CoV-2-6P-Mut7 + Fab J08 conformation 3. S-protein is labeled in gray, HC in dark blue, and LC in light blue. (*B*) *Side* and *Top* views of superimposed models of SARS-CoV-2-6P-Mut 2 (blue), SARS-CoV-2-6P-Mut 2 + FabJ08 (conformations 1 and 2; orange and green, respectively), and SARS-CoV-2-6P-Mut 7 + Fab J08 (conformation 3; red) reveal flexibility at the S1 domain that affects the opening at the apex. (*C*) Alignment of RBD-J08 X-ray structure onto protomer B of cryo-EM conformation 3 reveals a clash between the neighboring up-RBD and antibody. Clash sites are displayed as orange stars. (*D*) Alignment of RBD-J08 X-ray structure onto the ligand-free SARS-CoV-2-6P-Mut2 model reveals a clash with a neighboring RBD and its N343 glycan. Clash sites are displayed as orange stars. (*E*) The structure of the RBD (gray) across conformations 1 to 3 does not change when Fab J08 is bound. (*F*) CDRH3 (salmon) of Fab J08 exhibits more movement to accommodate binding to the RBD-up or -down conformations. On the other hand, CDRH2 (light orange) shows less movement across the different models.

For structural studies, we generated the following two constructs based on SARS-CoV-2-6P (six proline): one with the RBDs restricted to the down configuration by introduction of an interprotomer disulfide bond at positions C383 and C985 (Mut2) (31) and a second with unrestricted RBD movement but higher stability, containing an interprotomer disulfide bond at C705 and C883 (Mut7). Single-particle cryoelectron microscopy (cryo-EM) analysis of two complexes, SARS-CoV-2-6P-Mut2 + fragment antigen binding (Fab) J08 and SARS-CoV-2-6P-Mut7 + Fab J08, resulted in four cryo-EM maps and associated models, as follows: SARS-CoV-2-6P-Mut2 trimer alone (3.2 Å), SARS-CoV-2-6P-Mut2 + Fab J08 conformation 1 (3.4 Å), SARS-CoV-2-6P-Mut2 + Fab J08 conformation 2 (3.4 Å), and SARS-CoV-2-6P-Mut7 + Fab J08 conformation 3 (4.0 Å) (Fig. 2*A* and *SI Appendix*, Fig. S3 and Table S1). Although Fab J08 has nanomolar affinity to the trimer, the short incubation time during sample preparation for cryo-EM enabled reconstruction of a trimer with no antibody bound, serving as a comparator for our analysis with J08-bound structures. Lastly, we determined a 2.53-Å crystal structure of recombinant RBD in complex with Fab J08 to address details in the antibody–antigen interface (*SI Appendix*, Table S2).

In the SARS-CoV-2-6P-Mut2 + Fab J08 complex, we captured two different poses of Fab J08 through three-dimensional (3D) classification, which we call conformation 1 and conformation 2. In conformation 1, the J08 Fabs are further apart with a more closed apex (RBD more down), while conformation 2 has the Fabs closer together and a more open apex (RBD slightly open) (Fig. 2*A*). Superimposition of the S-protein models revealed movement in the S1 subunit, in comparison to minimal movement in the S2 subunit (Fig. 2*B*). Both conformations consist of three Fab molecules bound to a single S-protein trimer. In the SARS-CoV-2-6P-Mut7 + Fab J08 complex (conformation 3), Fab J08 bound to one RBD-up while the two other RBDs were down. Unlike the Mut2 complexes, most particles had a stoichiometry of only one Fab per S-protein trimer (Fig. 2*A*), although there was a small minority of particles with two Fabs bound in the negative-stain electron microscopy and cryoEM two-dimensional (2D) classes (*SI Appendix*, Fig. S4*A*). Alignment of the RBD-J08 X-ray structure onto the first cryo-EM conformation 3 down RBD (clockwise from the antibody-bound up RBD as viewed toward the viral membrane) reveals that a direct clash would occur with the up RBD (Fig. 2*C*). When aligned to the second down RBD (counterclockwise from the antibody-bound up RBD), a clash occurs between J08 CDRH3 and the neighboring down RBD and is likely exacerbated by the neighboring N343 glycan (*SI Appendix*, Fig. S4*B*). Finally, we find that the alignment of the RBD-J08 X-ray structure onto the ligand-free SARS-CoV-2-6P-Mut2 model reveals the same clash with neighboring RBD and glycan N343 (Fig. 2*D*), and highlights that even in the down-RBD models there is some degree of structural rearrangement and opening at the apex.

**Fig. 3.**
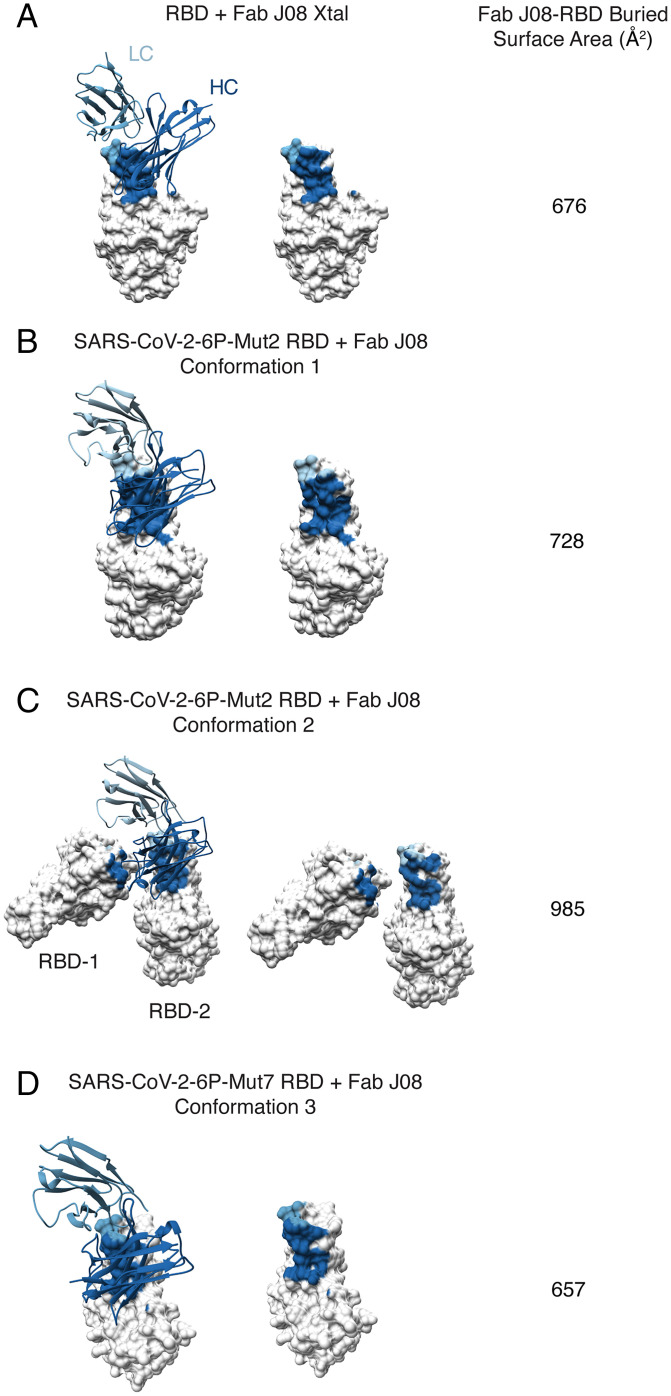
BSA and epitope footprint of J08 in X-ray and cryo-EM structures. J08 epitope footprint and calculated buried surface area in X-ray structure (*A*), cryo-EM conformation 1 (*B*), cryo-EM conformation 2 (*C*), and cryo-EM conformation 3 (*D*). Shown are surface representation of the RBD (gray) and ribbon representation of Fab J08 (HC, dark blue; LC, light blue).

Alignment of the RBD-antibody portion of each of the models revealed no major differences in the angle of the Fab relative to the RBD, and the epitope remained constant (Fig. 2*E*). However, more subtle differences were observed within the epitope–paratope interaction. The heavy chain complementarity-determining region 2 (CDRH2) loops were rigid, while the heavy chain CDRH3 loops were more variable, suggesting that CDRH2 was the anchoring interaction (Fig. 2*F*). These subtle differences might also be a result of differences in local resolution across the various datasets (*SI Appendix*, Fig. S3). Concomitant with these structural differences, we observed variable buried surface areas (BSAs) for Fab J08 bound to the three different conformations. Although J08 binds to the same epitope across the different conformations we observed that Fab J08 has the smallest footprint on the RBD in conformation 3 at 658 Å^2^, followed by conformation 1 at 728 Å^2^, and conformation 2 at 985 Å^2^ (Fig. 3). When not under the constraints of an intact protomer or trimer, the BSA between J08 and RBD based on the X-ray structure is 676 Å^2^, which is most similar to conformation 3.

J08 can accommodate the dynamic movement of the RBD with several key contacts (Fig. 3 *B*–*D* and *SI Appendix*, Table S3). CDRH2 appears to play an anchoring role and provides the most hydrogen bonds (including salt bridges) between antibody and RBD in conformations 1 and 2. In the up conformation 3, the antibody is tilted slightly, increasing the number of CDRH3 contacts, consistent with the X-ray structure of the RBD-Fab J08 complex (*SI Appendix*, Table S3). Accordingly, in conformations 1 and 3 and the X-ray structure, the heavy chain interface residues (defined as contributing greater than 5 Å^2^ BSA) all reside in CDRH2 and CDRH3 (*SI Appendix*, Tables S3 and S4). A portion of the interprotomer Fab contacts in conformation 2 are provided by CDRH1 (*SI Appendix*, Tables S3 and S4). On the contrary, the antibody light chain makes fewer contacts with the RBD and around 25% of the total BSA (Fig. 3 and *SI Appendix*, Table S3). CDRL1 and CDRL3 are part of the RBD interface in all three conformations, with only CDRL1 contributing hydrogen bond/salt bridge interactions (*SI Appendix*, Tables S3 and S4). We hypothesize that the unique ability to bind both RBD down and up is a contributing factor to the high potency of Fab J08.

### Molecular Description of the Epitope–Paratope Interface.

**Fig. 4.**
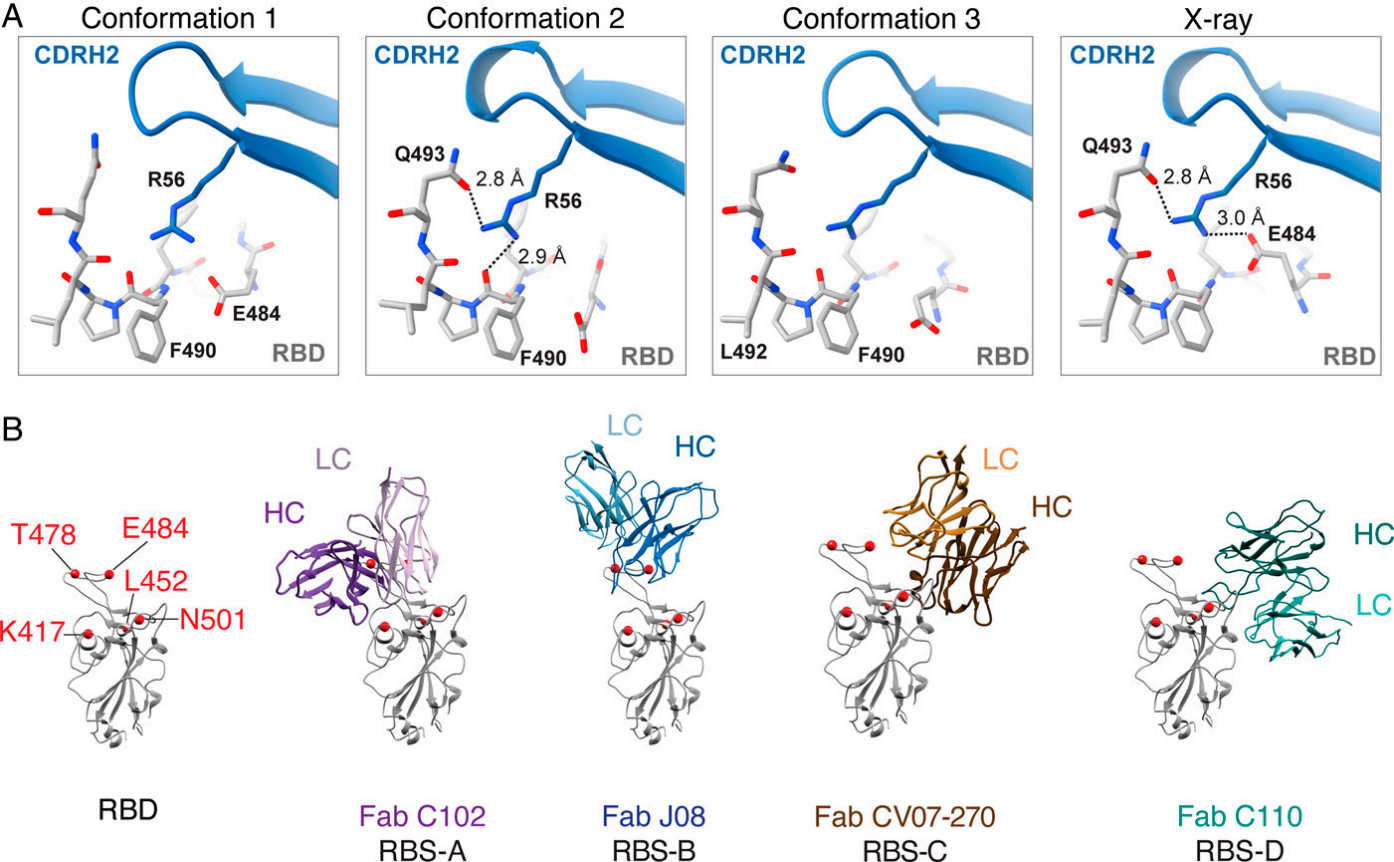
Molecular contacts and RBD epitope classification of Fab J08. (*A*) The key molecular environment between J08 CDRH2 residue R56 and RBD are highlighted, and predicted hydrogen bonds are represented as dashed lines with distances labeled. (*B*) Fab J08 belongs to the RBS-B class of antibodies in comparison to Fab C102, Fab CV07-270, and Fab C110 that belong to RBS-A, RBS-C, and RBS-D, respectively.

A commonality across the three binding conformations of J08 is residue R56 on the side chain of CDRH2 projecting toward the RBD, in a region bound by E484 and Q493 on opposite sides (Fig. 4*A*). In the X-ray structure of RBD-Fab J08, R56 is predicted to simultaneously form a salt bridge and hydrogen bond with the side chains of E484 and Q493, respectively (Fig. 4*A* and *SI Appendix*, Fig. S4*C*). Cryo-EM conformation 2 has the best-resolved map in this region and is predicted to also utilize a hydrogen bond with Q493, while swapping the E484 interaction with one to the F490 backbone carbonyl (Fig. 4*A* and *SI Appendix*, Fig. S4*D*). Antibody J08 is derived from IGHV1-69*02 and is hardly mutated relative to the germ line (96% amino acid sequence identity with only four mutations observed). Unexpectedly, all four mutations are in CDRH2 (corresponding to Kabat numbering 55 to 58) (*SI Appendix*, Fig. S4*E*). Relative to the germ line, the mutations include I56R, which is involved in key interactions as outlined above. The germ line I56 residue would not be capable of side-chain hydrogen bond interactions, suggesting this mutation was selected to increase affinity and/or specificity. Surprisingly, when we tested the R56I J08 IgG mutant using biolayer interferometry, we did not observe obvious changes in binding kinetics relative to wild-type J08 (*SI Appendix*, Fig. S1*F*). G55D and A57V do not directly interact with the RBD but appear to contribute to the overall stability and rigidity of CDRH2. D55 of CDRH2 forms a salt bridge with K73 of framework region 3 (FRH3), while V57, with its bulkier hydrophobic side chain relative to the germ line, points toward a region with several hydrophobic side chains, strengthening the interaction between beta strands of CDRH2 and FRH3 (*SI Appendix*, Fig. S4*F*). Based on the X-ray structure, N58M might also stabilize the local environment as it is part of a hydrophobic cluster that includes F486 (RBD), W47 (heavy chain [HC]), and L96 (light chain [LC]). All four of these mutations emphasize the importance of CDRH2 as the key anchoring point of J08 to the RBD. CDRH3, on the other hand, is predicted to form backbone hydrogen bonds with the side chains of RBD residues N487 and Y489, further strengthening the interaction of J08 to its relatively small epitope (*SI Appendix*, Fig. S4*H*). The light chain, derived from IGKV3-11, has two mutations relative to the germ line, one of which (L4M) might stabilize CDRL1, which is near the RBD ridge (*SI Appendix*, Fig. S4 *E* and *G*).

### Fab J08 Does Not Interact with Most Mutations Found in VoCs.

Since Fab J08 binds high on the RBD ridge, it is far less susceptible to the commonly mutated residues in the receptor binding site (RBS) in the emerging VoCs. As previously described (32), the RBS is subdivided into four epitopes, delineated as RBS-A, -B, -C, and -D. Fab J08 belongs to the RBS-B class, in comparison to other antibodies such as Fab C102 (RBS-A), Fab CV07-270 (RBS-C), and C110 (RBS-D) (Fig. 4*B*). Many of the commonly mutated residues exist in these epitopes, thus increasing the odds of viral escape (Fig. 4*B* and *SI Appendix*, Fig. S5). For example, K417 resides in RBS-A, E484 in RBS-B, L452 and E484 in RBS-C, and N501 in between RBS-A and RBS-D. Studies have shown that the binding potency and neutralization capacity of several previously isolated mAbs are severely reduced or abrogated in the presence of one of these point mutations (6).

N501Y, a mutation found in the Alpha, Beta, Gamma, and Omicron variants, is not part of the J08 interface, explaining why neutralization against the Alpha variant is unaffected. Common to the Beta, Gamma, and Omicron variants are mutations at position 417 (K417N in Beta and Omicron, K417T in Gamma). K417 is at the interface with J08 in conformations 1 and 3 and the X-ray structure, contributing a hydrogen bond via its side chain amine with CDRH3 in conformations 1 and 3 (*SI Appendix*, Fig. S4*D* and Tables S3 and S4). Since neutralization against Beta and Gamma variants is unaffected, we conjecture that this interaction is not critical and becomes redundant with additional CDRH3 residues involved in the RBD interaction. The E484K mutation is shared among the Alpha, Beta, and Gamma variants, while Omicron presents a shorter and hydrophobic E484A mutation at the same site. E484, as mentioned, is predicted to form a salt bridge with R56 of CDRH2 in some of the conformations (*SI Appendix*, Table S3), so introducing a basic residue in its place might affect binding. However, neutralization and a negative-stain EM complex of J08 and Gamma S-protein suggests otherwise (Fig. 1 and *SI Appendix*, Fig. S5*D*), perhaps due to the versatility of R56 finding alternate contacts (e.g., with RBD backbone carbonyls in conformation 3). Finally, the Delta variant has an L452R mutation, and shares an additional T478K mutation with Omicron, and while T478 appears at the LC interface, neither residue has a measured interaction with J08 in our structures (*SI Appendix*, Fig. S5 and Table S4).

The more recent Omicron VOC has over 35 mutations in the S-protein, with 15 residing in the RBD. As described above, specific point mutations shared with other VOCs do not appear to impede neutralization, but our assays reveal a sharp decrease in neutralization and high off-rate of J08 against Omicron itself. To address whether the overall epitope is altered on the Omicron surface, we generated a ∼6-Å cryo-EM reconstruction of J08 in complex with the Omicron-CoV-2-6P S-protein (*SI Appendix*, Fig. S5 and Table S1). We observe one Fab bound to a subpopulation of S-protein, and a comparison to our SARS-CoV-2-6P D614G (Wuhan) S-protein-J08 complex (conformation 3) map suggests that the overall epitope footprint is not changed. However, Omicron contains a Q493R mutation not found in other VOCs. Our models of J08 in complex with the SARS-CoV-2 6P D614G (Wuhan) strain S-protein/RBD suggest an interdependence between E484 and Q493 of RBD with J08 CDRH2 R56, and we infer that a mutation of the 484 and 493 sites in Omicron is the primary factor leading to decreased sensitivity of J08 against this variant.

**Fig. 5.**
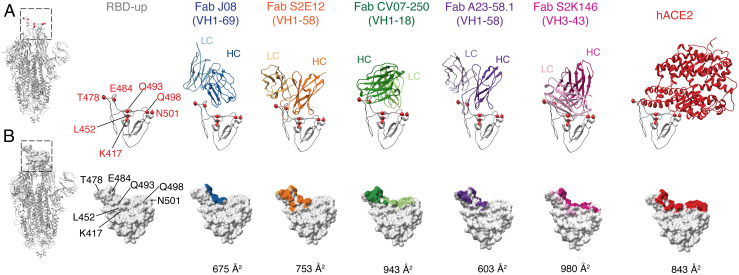
Epitope footprint comparison of Fab J08 to similar antibodies and hACE2. (*A*) Fab J08 compared to antibodies S2E12, CV07-250, A23-58.1, and S2K146 that share similar angles of approach and bind high on the RBD ridge, thereby allosterically inhibiting hACE2 binding. Commonly mutated residues K417, L452, T478, E484, Q493, Q498, and N501 are shown as a point of reference and orientation of the RBD. (*B*) Surface representation of the RBD with antibody contacts colored on the surface and calculated BSA values (Å^2^) reveal that Fab J08 has a small footprint and is therefore less susceptible to escape mutations.

RBS-B antibodies that bind high on the RBD-ridge, which share a similar angle of approach as Fab J08 and sterically block binding of hACE2, include S2E12 (33), CV07-250 (34), A23-58.1 (35), and S2K146 (36) (Fig. 5*A*). In comparison to J08, S2E12, and A23-58.1, the HCs and LCs of CV07-250 and S2K146 are rotated ∼90 degrees clockwise, thereby expanding the footprint on the RBD and using more LC contacts than just primarily HC contacts. In fact, this rotation and larger footprint are remarkably similar to hACE2 (Fig. 5*B*). In decreasing order, the RBD-antibody/receptor BSA is largest for S2K146 (980 Å^2^), CV07-250 (943 Å^2^), ACE2 (843 Å^2^), S2E12 (753 Å^2^), J08 (675 Å^2^, based on the RBD-Fab J08 X-ray structure), and A23-58.1 (603 Å^2^). S2E12 and A23-58.1 are most similar to J08 based on epitope, as the antibodies make most of their contacts with the RBD via the HC and can also neutralize variants containing the E484K/D614G and E484Q/D614G/Q779H mutations. A difference is that the CDRH3 of S2E12 and A23-58.1 are nestled more against the RBD ridge, in closer proximity to the 477/478 residues that are mutated in some VOCs. However, the CDRH2 of each antibody appears farther away from E484/Q493 than J08, perhaps making them less susceptible to mutations at those sites. Like J08, S2E12 and A23-58.1 would require a partially open Spike to avoid clashing with a neighboring RBD and glycan N343. While the Omicron variant has affected many mAbs targeting the RBD, including J08, it is noteworthy that 45% of RBD residues involved in the RBD-ACE2 interface are also part of the J08 interface, and these residues in turn account for 77% of the RBD-J08 interface (*SI Appendix*, Fig. S6), suggesting that escape mutations to J08 that do not also negatively impact viral fitness are rare.

## Discussion

We used a combination of cell-based assays and structural biology to better understand the remarkable potency and resistance to many VoCs by mAb J08. Neutralization assays using authentic virus and pseudovirus revealed that Fab J08 was able to neutralize VoCs at a low nanomolar affinity and outperform similar RBD-binding competitor antibodies, such as REGN10987/10933, S309, COV2.2196, and LY-COV016 in the low-nanomolar range. The Omicron variant presents a challenge to J08, but we still found the antibody capable of neutralization, again outperforming other monoclonals that are escaped by this VOC. Using cryo-EM and X-ray crystallography experiments, we showed that J08 can bind to multiple conformations of the S-protein with the RBD in either the up or partially down position and its smaller epitope footprint is distant from many of the common mutations found in the VOCs. E484, while on the edge of the epitope and a potential hydrogen bond or salt bridge partner, does not appear to be straddled by the antibody in a way that would create a clash in the E484K variant. Nonetheless, when coupled with a Q493H mutation, as the case in the Omicron VOC, both binding affinity and neutralization are negatively impacted. mAb J08 is currently being evaluated in clinical trials to test its utility as an important intervention therapeutic for moderate-to-severe COVID-19. The very high potency and ability to resist many escape mutations are critical properties for the next generation of SARS-CoV-2 mAb therapeutics.

## Materials and Methods

### ELISA.

High-binding 96-well ELISA plates (Costar, Corning) were coated overnight with 250 ng/well of purified recombinant SARS-CoV-2 proteins. After being washed with 0.05% Tween 20–phosphate-buffered saline (PBST), plates were blocked for 2 h with 2% bovine serum albumin, 1 mM ethylenediaminetetraacetic acid (EDTA), and PBST (blocking buffer); washed; and incubated with purified monoclonal IgG antibodies at 10 µg/mL and 7 consecutive 1:4 dilutions in PBS. After the PBST washing, the plates were incubated with goat horseradish peroxidase (HRP)-conjugated anti-human IgG antibodies for 1 h (Jackson ImmunoReseach, 0.8 µg/mL final in blocking buffer) and analyzed by adding 100 µL of HRP chromogenic substrate (ABTS solution, Euromedex) after the washing steps. For competition experiments of RBD binding to ACE-2, ELISA plates were coated overnight with 250 ng/well of purified ACE-2 ectodomain. After the PBST wash step, plates were blocked for 2 h with blocking buffer, washed with PBST, and incubated with purified monoclonal IgG antibodies at 10 µg/mL and 7 consecutive 1:2 dilutions in PBS in the presence of biotinylated RBD proteins at 0.5 µg/mL After being washed, the plates were incubated for 30 min with HRP-conjugated streptavidin (BD Biosciences) and analyzed by adding 100 µL of the HRP chromogenic substrate (ABTS solution, Euromedex). Optical densities were measured at 405 nm (OD_405nm_), and background values, assessed by incubation of PBS alone in coated wells, were subtracted. Experiments were performed using a HydroSpeed microplate washer and Sunrise microplate absorbance reader (Tecan Männedorf).

### SARS-CoV-2 Authentic Virus Neutralization Assay.

All SARS-CoV-2 authentic virus neutralization assays were performed in the biosafety level 3 (BSL3) laboratories at Toscana Life Sciences in Siena (Italy), Vismederi Srl, Siena (Italy), and Institute Pasteur, Paris (France). The BSL3 laboratories are approved by a Certified Biosafety Professional and inspected every year by local authorities. Two different approaches were used to evaluate the neutralization activity of J08 against SARS-CoV-2 and emerging variants and the neutralization breadth of tested antibodies. The first method is the cytopathic effect (CPE)-based neutralization assay described by Andreano and colleagues (13), while the second method is a S-fuse neutralization assay previously described by Planas et al. (26). Briefly, the CPE-based neutralization assay reports on the coincubation of mAbs with a SARS-CoV-2 solution containing 100 median tissue culture infectious dose (TCID_50_) of virus and after 1 h incubation at 37 °C and 5% CO_2_. The mixture was then added to the wells of a 96-well plate containing a subconfluent Vero E6 cell monolayer. Plates were incubated for 3 d at 37 °C in a humidified environment with 5% CO_2_ and then examined for CPE by means of an inverted optical microscope. As for the S-fuse neutralization assay, U2OS-ACE2 GFP1-10 or GFP 11 cells, also termed S-Fuse cells, emit fluorescence when they are productively infected by SARS-CoV-2 (26, 37). Cells were tested negative for mycoplasma. Cells were mixed (1:1 ratio) and plated at 8 × 10^3^ per well in a μClear 96-well plate (Greiner Bio-One). SARS-CoV-2s were incubated with mAbs for 15 min at room temperature and added to S-Fuse cells. After 18 h, cells were fixed with 2% paraformaldehyde, washed, and stained with Hoechst solution (1:1,000 dilution; Invitrogen). Images were acquired with an Opera Phenix high-content confocal microscope (PerkinElmer). The GFP area and the number of nuclei were quantified using the Harmony software (PerkinElmer). The percentage of neutralization was calculated using the number of syncytia as a value with the following formula: 100 × (1 – (value with mAb – value in “non-infected”)/(value in “no mAb” – value in “non-infected”)). We previously reported a correlation between neutralization titers obtained with the S-Fuse assay and a pseudovirus neutralization assay (38).

### SARS-CoV-2 Variants for CPE-MN and S-fuse Neutralization Assays.

The SARS-CoV-2s used to perform the CPE-MN neutralization assay were D614G(EVAg Cod: 008V-04005), B.1.1.7 (INMI GISAID accession number: EPI_ISL_736997), B.1.351 (EVAg Cod: 014V-04058), P.1 (EVAg CoD: 014V-04089), B.1.617.2 (ID: EPI_ISL_2029113), and B.1.1.529 (ID: EPI_ISL_6794907). The SARS-CoV-2s used to perform the S-fuse neutralization assay were D614G, B.1.1.7, B.1.351, and B.1.617.2, and their sequences were deposited on GISAID, with the following identifiers: D614G: EPI_ISL_414631, B.1.1.7: EPI_ISL_735391, B.1.1.351: EPI_ISL_964916, and B.1.617.2: ID: EPI_ISL_2029113 (27).

### HEK293TN-hACE2 Cell Line Generation.

An HEK293TN-hACE2 cell line was generated by lentiviral transduction of HEK293TN cells as described in Notarbartolo S. et al. (39). Briefly, HEK293TN cells were obtained from System Bioscience. Lentiviral vectors were produced following a standard procedure based on calcium phosphate cotransfection with third generation helper and transfer plasmids. The following helper vectors were used (gifts from Didier Trono, School of Life Sciences, Ecole Polytechnique Fédérale de Lausanne (EPFL), Lausanne, Switzerland): pMD2.G/VSV-G (Addgene #12259), pRSV-Rev (Addgene #12253), and pMDLg/pRRE (Addgene #12251). The transfer vector pLENTI_hACE2_HygR was obtained by cloning hACE2 from pcDNA3.1-hACE2 (a gift from Fang Li, Department of Veterinary and Biomedical Sciences, University of Minnesota, Saint Paul, MN, Addgene #145033) into pLenti-CMV-GFP-Hygro (a gift from Eric Campeau & Paul Kaufman, Program in Gene Function and Expression, University of Massachusetts Medical School, Worcester, MA, Addgene #17446). hACE2 complementary DNA was amplified by PCR and inserted under the CMV promoter of the pLenti-CMV-GFP-Hygro after GFP excision with XbaI and SalI digestion. pLENTI_hACE2_HygR is now available through Addgene (Addgene #155296). After transduction with the hACE2 lentiviral vector, cells were subjected to antibiotic selection with hygromycin at 250 μg/mL. The expression of hACE2 cells was confirmed by flow cytometry staining using an anti-hAce2 primary antibody (AF933; R&D system) and rabbit anti-goat IgG secondary antibody (Alexa Fluor 647). HEK293TN-hACE2 cells were maintained in Dulbecco's Modified Eagle Medium (DMEM) and supplemented with 10% FBS, 1% glutamine, 1% penicillin/streptomycin, and 250 μg/mL hygromicin (GIBCO), and the expression of hACE2 was found to be stable after multiple passages.

### Production of SARS-CoV-2 Pseudoparticles Based on Lentiviral Vectors.

To generate SARS-CoV-2 lentiviral pseudotype particles, 5 × 10^6^ HEK-293TN cells were plated in a 15-cm dish in complete DMEM. The following day, 32 µg of reporter plasmid pLenti CMV-GFP-TAV2A-LUC Hygro, 12.5 mg of pMDLg/pRRE (Addgene #12251), 6.25 mg of pRSV-Rev (Addgene #12253), and 9 µg pcDNA3.1_ S-protein_del19 were cotransfected following a calcium phosphate transfection. pcDNA3.1_S-protein_del19 was generated by deletion of the last 19 amino acids of S-protein starting from pcDNA3.1-SARS2-S-protein (a gift from Fang Li, Addgene plasmid #145032) and is now available through Addgene (Addgene #155297). pLenti CMV-GFP-TAV2A-LUC Hygro was generated from pLenti CMV GFP Hygro (Addgene #17446) by addition of T2A-Luciferase by PCR cloning. At 12 h before transfection, the medium was replaced with complete Iscove medium. At 30 h after transfection, the supernatant was collected, clarified by filtration through 0.45-μm pore-size membranes, and concentrated by centrifugation for 2 h at 20,000 rpm using an SW32Ti rotor. Viral pseudoparticle suspensions were aliquoted and stored at −80 °C.

### SARS-CoV-2 Pseudovirus Neutralization Assay.

Pseudovirus neutralization assays were carried out as previously described (40). Briefly, HEK293TN-hACE2 cells were plated at 10^4^ cells/well in white 96-well plates in complete DMEM. At 24 h later, cells were infected with a 0.1 multiplicity of infection (MOI) of SARS-CoV-2 pseudoparticles that were previously incubated with a serial dilution of mAb. In particular, mAbs under test were serially diluted fivefold in PBS in order to obtain a seven-point dose–response curve (plus PBS as an untreated control). Thereafter, 5 µL of each dose–response curve point was added to 45 µL of medium containing SARS-CoV-2 pseudoparticles adjusted to contain 0.1 MOI. After incubation for 1 h at 37 °C, 50 µL of a mAb/SARS-CoV-2 pseudoparticle mixture was added to each well and plates were incubated for 24 h at 37 °C. Each point was assayed in triplicate. After 24 h of incubation, cell infection was measured by a luciferase assay using the Bright-Glo luciferase system (Promega) and an Infinite F200 plate reader (Tecan) to read the luminescence. Obtained relative light units were normalized to controls, and dose–response curves were generated by nonlinear regression curve fitting with GraphPad Prism to calculate neutralization dose 50 (ND_50_).

### Expression and Purification of SARS-CoV-2 S-protein in the Prefusion Conformation.

Mutagenesis was performed on the SARS-CoV-2-6P plasmid to include S383C and D985C for the SARS-CoV-2-6P-Mut2 construct and V705C and T883C for the SARS-CoV-2-6P-Mut7 construct. The Omicron SARS-CoV-2 plasmid contained only the 6P mutations (Omicron-CoV-2-6P). Expression of the SARS-CoV-2-6P-Mut2, SARS-CoV-2-6P-Mut7, or Omicron-CoV-2-6P S-protein was performed by incubating 0.5 mg of DNA with 1.5 mg of polyethylenimine for 20 min. The mixture was placed into 1 L of HEK293F cells (Thermo Fisher), incubated for 6 d at 37 °C with 8% CO_2_ and shaken at 125 rpm. After cell harvest, the supernatant was passed over a StrepTactin XT 4FLOW column (IBA Lifesciences), washed with buffer W (100 mM Tris-Cl [pH 8], 150 mM NaCl, 1 mM EDTA), and eluted with buffer BXT (100 mM Tris-Cl [pH 8], 150 mM NaCl, 1 mM EDTA, 50 mM biotin). The eluant was then size exclusion purified over a Superose 6 Increase-16/600 pg, 120-mL column (Cytiva). Purified trimers were buffer exchanged back into buffer W using a 100-kDa concentrator (Amicon).

### Sample Vitrification for Cryo-EM.

SARS-CoV-2-6P-Mut2 was incubated with a threefold molar excess of Fab J08 at room temperature for 5 min. The final concentration of the complex was 3 mg/mL. To aid with sample dispersal on the grid, the complex was briefly incubated with n-dodecyl-B-D-maltoside (final concentration, 0.06 mM; Anatrace) and deposited on plasma-cleaned Quantifoil 1.2/1.3 4C grids. A Thermo Fisher Vitrobot Mark IV set to 4 °C, 100% humidity, 6-s wait time, and a 3-s blot time was used for the sample vitrification process. SARS-CoV-2-6P-Mut7 or Omicron-CoV-2-6P were incubated with a threefold molar excess of Fab J08 at room temperature for 30 min. The sample vitrification process was as described above except that the detergent for SARS-CoV-2-6P-Mut7 was fluorinated octyl maltoside (final concentration of 0.02%, wt/vol; Anatrace) and the grids were UltrAuFoil 1.2-1.3 3C. For Omicron-CoV-2-6P, the detergent used was lauryl maltose neopentyl glycol (final concentration of 0.005 mM; Anatrace) and the grids were Quantifoil 1.2/1.3 2C.

### Cryo-EM Data Collection.

Datasets for both SARS-CoV-2-6P-Mut2 and SARS-CoV-2-6P-Mut7 complexes were collected at 36,000× magnification on a Thermo Fisher Talos Arctica (200 keV, 1.15-Å pixel size) electron microscope with a 4k by 4k Gatan K2 Summit direct electron detector. Data collection was automated with the Leginon software (41), and raw micrographs were stored in the Appion database (42). For the Omicron-CoV-2-6P complex, data collection was performed on a Thermo Fisher Glacios (200 keV, 0.57-Å pixel size) using a Thermo Fisher Falcon 4 direct electron detector and Thermo Fisher EPU 2 software.

For the SARS-CoV-2-6P-Mut2 + Fab J08 complex, a total of 2,325 micrographs were collected with a total dose of 50 e-/Å^2^ fractionated over 48 frames, with each frame receiving a dose rate of 5.5 electrons per pixel per second. A defocus range of −0.2 μm to −2.4 μm was used. For the SARS-CoV-2-6P-Mut7 + Fab J08 complex, 4,090 micrographs were collected with a total dose of 50 e-/Å^2^ fractionated over 50 frames, with each frame receiving a dose rate of 5.2 electrons per pixel per second. In this case, a defocus range of −0.5 μm to −2.0 μm was used. For the Omicron-CoV-2-6P + Fab J08 complex, 4,146 micrographs were collected with a total dose of 49 e-/Å^2^ fractionated over 40 frames, with each frame receiving a dose rate of 4.4 electrons per pixel per second. A defocus range of −0.7 μm to −1.4 μm was used.

### Cryo-EM Data Processing, Model Building, and Refinement.

The micrograph movie frames from the SARS-CoV-2-6P-Mut2 and SARS-CoV-2-6P-Mut7 datasets were aligned and dose weighted with MotionCorr2 (43). Aligned frames were imported into cryoSPARC v3.2 (44) where the contrast transfer function (CTF) was estimated using Patch CTF. For the Omicron-CoV-2-6P dataset, the Patch Motion Correction job of cryoSPARC Live was used for alignment and dose weighting of movies (44). Particles were picked using templates (created from an initial round of 2D classification after automated picking), extracted, and subjected to multiple rounds of 2D classification for cleaning. For the SARS-CoV-2-6P-Mut2 and SARS-CoV-2-6P-Mut7 datasets, an apo (unliganded) S-protein was imported for 3D classification (heterogeneous refinement) and the best classes were further refined. To further improve the resolution, the maps were subjected to global and local CTF refinements and 3D variability analyses. Final refinements were performed using the nonuniform refinement feature (45). The Omicron-CoV-2-6P dataset particles were exported to Relion 3.1 (46) and downscaled to 1.14 Å/pixel to reduce computational demands resulting from a large box size. After an additional round of 2D classification and automated 3D refinement, the particles were subjected to C3 symmetry expansion. A 40-Å-diameter spherical mask was placed over the RBD/Fab of a single protomer and iterative rounds of 1) alignment-free 3D classification and 2) 3D refinement with restricted search angles using a mask over the trimer core and a single RBD+Fab were performed to isolate J08-bound particles. A summary of data collection and processing statistics can be found in *SI Appendix*, Table S1.

Initial models were generated by fitting Spike coordinates from Protein Data Bank (PDB) 6vsb and the RBD-J08 X-ray structure (see below) into the cryo-EM maps using University of California San Francisco Chimera (47). Several rounds of iterative manual and automated model building and relaxed refinement were performed using Coot 0.9.4 (48) and Rosetta (49). Models were validated using EMRinger (50) and MolProbity (51) as part of the Phenix software suite (52). Kabat numbering was applied to the antibody Fab variable LCs and HCs using the Abnum antibody numbering server (53). Final refinement statistics and PDB deposition codes for generated models can be found in *SI Appendix*, Table S1. Buried surface area calculations and distance measurements were performed using PDBePISA (54).

### Crystallization and X-Ray Structure Determination.

The J08 Fab complexed with SARS-CoV-2 RBD was formed by mixing each of the protein components in an equimolar ratio and incubating overnight at 4 °C. A total of 384 conditions of the JCSG Core Suite (Qiagen) were used for setting up trays for the complex (6 mg/mL) on the robotic CrystalMation system (Rigaku) at Scripps Research. Crystallization trials were set up by the vapor diffusion method in sitting drops containing 0.1 μL of protein complex and 0.1 μL of reservoir solution. Crystals appeared on day 3, were harvested on day 7, pre-equilibrated in cryoprotectant containing 10% ethylene glycol, and then flash cooled and stored in liquid nitrogen until data collection. Diffraction data were collected at cryogenic temperature (100 K) at beamline 12-1 of the Stanford Synchrotron Radiation Lightsource (SSRL) and processed with HKL2000 (55). Diffraction data were collected from crystals grown in drops containing 17% (wt/vol) polyethylene glycol 4000, 15% (vol/vol) glycerol, 8.5% (vol/vol) isopropanol, and 0.085 M sodium Hepes (pH 7.5). The X-ray structures were solved by molecular replacement (MR) using PHASER (56) with MR models for the RBD and Fab from PDB 7JMW (57). Iterative model building and refinement were carried out in COOT (48) and PHENIX (52), respectively. X-ray data collection and structural refinement statistics can be found in *SI Appendix*, Table S2.

### Biolayer Interferometry.

Anti-human IgG Fc Capture (AHC) biosensors (ForteBio) were used to assess the binding affinities of J08 IgG and J08 R56I IgG with SARS-2 CoV 6P D614G and Omicron 6P in kinetics buffer (PBS + 0.02% Tween-20 + 0.1% bovine serum albumin) on an Octet RED96 instrument (ForteBio). Initially, the biosensors were soaked in kinetics buffer prior to loading the IgGs to a threshold of 1.0 nm. The biosensors were dipped into kinetics buffer for a second baseline and then dipped into wells containing S-protein at twofold dilutions with seven different concentrations (31.3, 15.6, 7.3, 3.65, 1.82, 0.91, and 0.45 nM for SARS-CoV-2-6P D614G (Wuhan) and 1,000, 500, 250, 125, 62.5, 31.3, and 15.6 nM for Omicron-CoV-2-6P) to measure association. Biosensors were then dipped into wells containing kinetics buffer to measure dissociation. Data were reference subtracted based on a kinetics buffer baseline and aligned to each other using Octet Data Analysis software (ForteBio). Curve fitting was performed using a 1:1 binding model and data for the seven concentrations of S-protein, but only those that fit the raw data best were included, which are labeled with a red dashed line. On-rate, off-rate, and K_D_ values were determined with a global fit. Data were exported from the ForteBio Data Analysis software and plotted using GraphPad Prism.

## Supplementary Material

Supplementary File

## Data Availability

The data needed to evaluate conclusions in this paper are present in both the paper itself and/or in *SI Appendix*. The cryo-EM maps have been deposited in the Electron Microscopy Data Bank (EMDB) with accession codes EMD-24876 (SARS-CoV-2-6P-Mut2), EMD-24877 (SARS-CoV-2-6P-Mut2 + Fab J08 conformation 1), EMD-24878 (SARS-CoV-2-6P-Mut2 + Fab J08 conformation 2), EMD-24879 (SARS-CoV-2-6P-Mut7 + Fab J08), and EMD-26389 (Omicron SARS-CoV-2-6P + Fab J08) and the atomic models deposited in the Protein Data Bank (PDB) with accession codes 7s6i (SARS-CoV-2-6P-Mut2), 7s6j (SARS-CoV-2-6P-Mut2 + Fab J08 conformation 1), 7s6k (SARS-CoV-2-6P-Mut2 + Fab J08 conformation 2), and 7s6l (SARS-CoV-2-6P-Mut7 + Fab J08). The coordinate and structure factor of the crystal structure of SARS-CoV-2 RBD in complex with Fab J08 has been deposited in the PDB with accession code 7sbu.
