# Supplementary material for: Structural insights of a highly potent pan-neutralizing SARS-CoV-2 human monoclonal antibody
Source: Proc Natl Acad Sci U S A. 2022 May 12;119(20):e2120976119. doi: 10.1073/pnas.2120976119 (PMC9171815; doi:10.1073/pnas.2120976119)
Supplement: Supplementary File [file pnas.2120976119.sapp.pdf]

PNAS
www.pnas.org

Supplementary Information for

Structural insights of a highly potent pan-neutralizing SARS-CoV-2 human monoclonal antibody

Jonathan L. Torres[1]†, Gabriel Ozorowski[1]†, Emanuele Andreano[2], Hejun Liu[1], Jeffrey Copps[1], Giulia Piccini[3], Lorena Donnici[5], Matteo Conti[5], Cyril Planchais[6], Delphine Planas[7,8], Noemi Manganaro[2], Elisa Pantano[2], Ida Paciello[2], Piero Pileri[2], Timothée Bruel[7,8], Emanuele Montomoli[3,4,9], Hugo Mouquet[6], Olivier Schwartz[7,8], Claudia Sala[2], Raffaele De Francesco[5,12], Ian A. Wilson[1,10], Rino Rappuoli[2,11], Andrew B. Ward[1]*

[1]Department of Integrative Structural and Computational Biology, The Scripps Research Institute, La Jolla, CA 92037, USA

[2]Monoclonal Antibody Discovery (MAD) Lab, Fondazione Toscana Life Sciences, Siena, Italy

[3]VisMederi S.r.l, Siena, Italy

[4]VisMederi Research S.r.l., Siena, Italy

[5]INGM, Istituto Nazionale Genetica Molecolare "Romeo ed Enrica Invernizzi", Milan, Italy.

[6]Laboratory of Humoral Immunology, Department of Immunology, Institut Pasteur, INSERM U1222, Paris, France

[7]Virus and Immunity Unit, Department of Virology, Institut Pasteur, CNRS UMR 3569, Paris, France.

[8]Vaccine Research Institute, Creteil, France

[9]Department of Molecular and Developmental Medicine, University of Siena, Siena, Italy

[10]The Skaggs Institute for Chemical Biology, The Scripps Research Institute, La Jolla, CA 92037, USA
[11]Department of Biotechnology, Chemistry and Pharmacy, University of Siena, Siena, Italy
[12]Department of Pharmacological and Biomolecular Sciences DiSFeB, University of Milan, Milan, Italy

†These authors contributed equally to this work
*Correspondence: Andrew B. Ward. **Email:** andrew@scripps.edu

This PDF file includes:

Figures S1 to S6
Tables S1 to S4
SI References

SI Figures and Tables

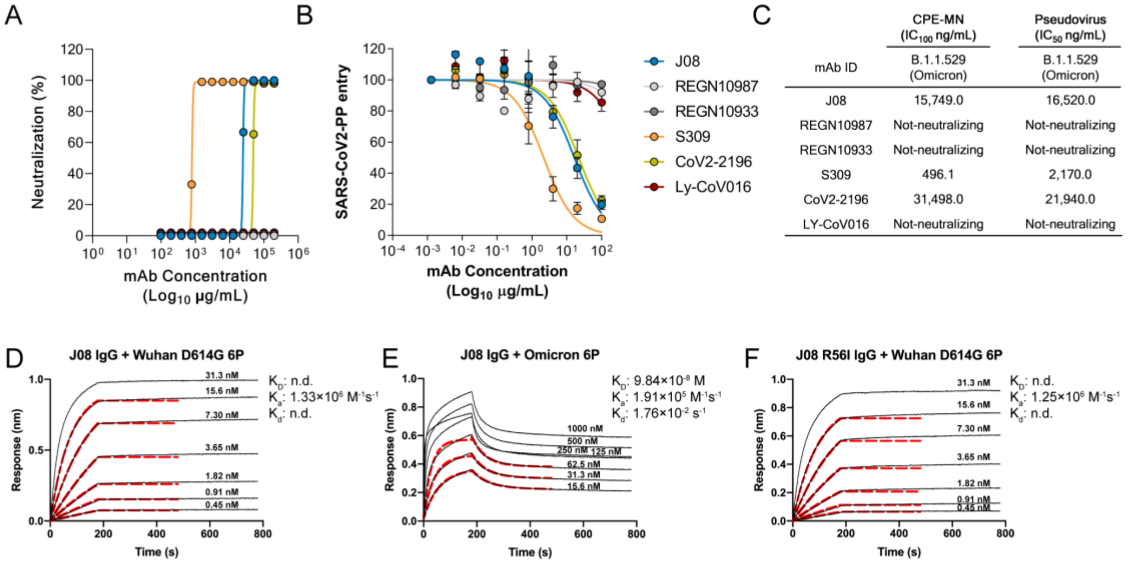

mAb ID	CPE-MN (IC_{100} ng/mL) B.1.1.529 (Omicron)	Pseudovirus (IC_{50} ng/mL) B.1.1.529 (Omicron)
J08	15,749.0	16,520.0
REGN10987	Not-neutralizing	Not-neutralizing
REGN10933	Not-neutralizing	Not-neutralizing
S309	496.1	2,170.0
CoV2-2196	31,498.0	21,940.0
LY-CoV016	Not-neutralizing	Not-neutralizing

Fig. S1. Neutralization activity and binding kinetics against Omicron VOC. (**A**) CPE-MN neutralization activity against SARS-CoV-2 B.1.1.529 for J08, REGN10987, REGN10933, S309, CoV2-2196 and LY-CoV016. (**B**) Neutralization activity against SARS-CoV-2 B.1.1.529 pseudoviruses for J08, REGN10987, REGN10933, S309, CoV2-2196 and LY-CoV016. (**C**) Summary of the IC_{100} and IC_{50} results obtained for all neutralization assays. (**D-F**) Biolayer interferometry of J08 IgG + SARS-CoV-2-6P D614G (Wuhan), J08 IgG + Omicron-CoV-6P, and J08 R56I IgG + SARS-CoV-2-6P D614G (Wuhan). Fit curves used for calculating kinetics values shown as red dashes. n.d.: not determined.

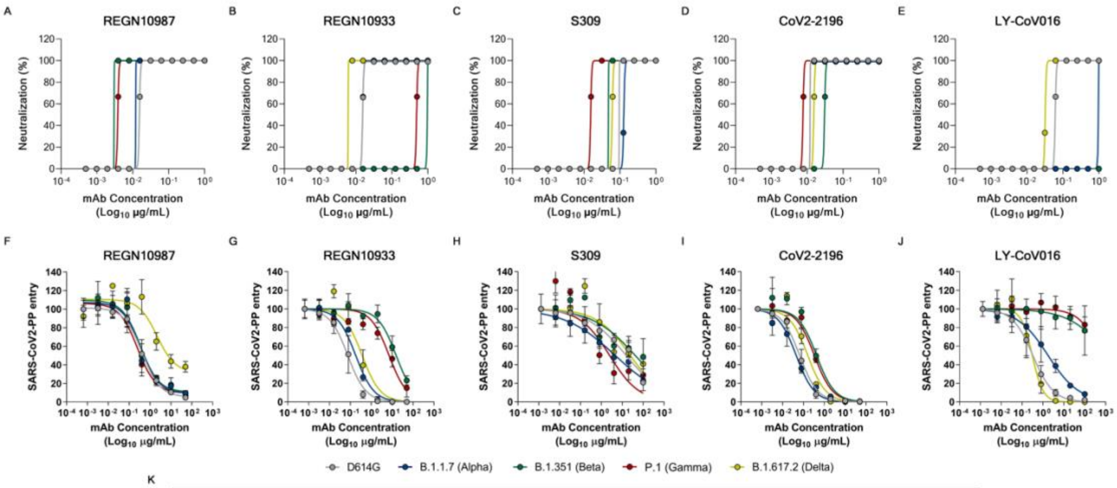

K

mAb ID	CPE-MN (IC_{100} ng/mL) D614G	B.1.1.7 (Alpha)	B.1.351 (Beta)	P.1 (Gamma)	B.1.617.2 (Delta)	Pseudovirus (IC_{50} ng/mL) D614G	B.1.1.7 (Alpha)	B.1.351 (Beta)	P.1 (Gamma)	B.1.617.2 (Delta)
REGN10987	24.6	19.5	4.9	3.1	19.7	41.0	37.0	35.0	25.0	2,154
REGN10933	24.6	24.6	1,574	787.4	7.8	75.0	179	15,460	7,120	365
S309	156.2	248.0	78.1	24.6	78.7	15,000	4,600	41,000	2,500	24,000
CoV2-2196	19.5	19.5	49.2	12.3	19.7	66.0	45.0	425.0	335.0	161.0
LY-CoV016	124.0	1,574	Not-neutralizing	Not-neutralizing	49.6	326.0	1,744	Not-neutralizing	Not-neutralizing	305.0

Fig. S2. Neutralization activity of competitor mAbs. (**A-E**) CPE-MN neutralization activity against SARS-CoV-2 D614G, B.1.1.7, B.1.351, P.1, and B.1.617.2 for REGN10987 (**A**), REGN10933 (**B**), S309 (**C**), CoV2-2196 (**D**) and LY-CoV016 (**E**). (**F-J**) Neutralization activity against SARS-CoV-2 D614G, B.1.1.7, B.1.351, P.1, and B.1.617.2 pseudoviruses for REGN10987 (**F**), REGN10933 (**G**), S309 (**H**), CoV2-2196 (**I**) and LY-CoV016 (**J**). (**K**) Summary of the IC_{100} and IC_{50} results obtained for all neutralization assays.

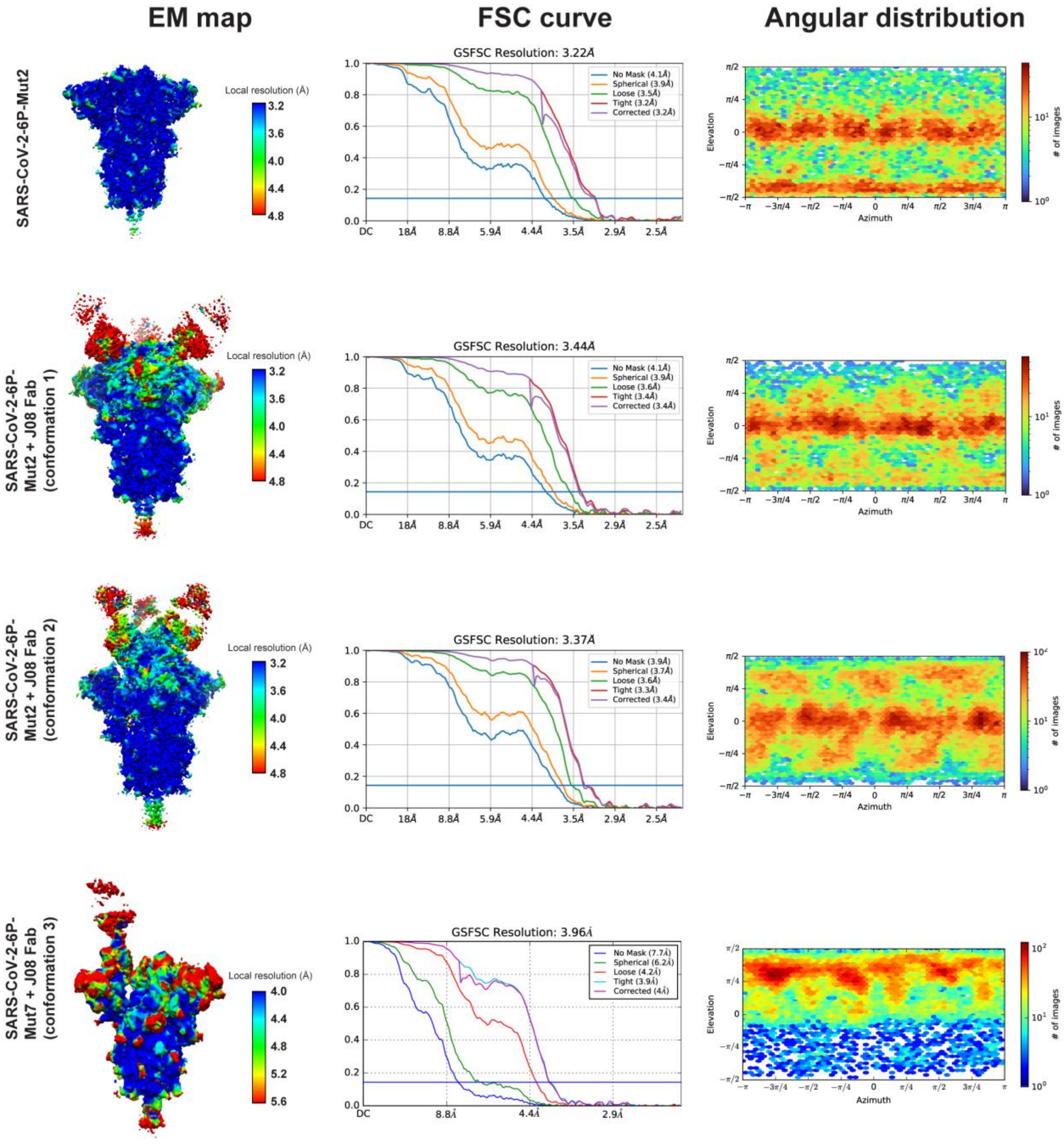

Fig. S3. Cryo-EM resolution estimates and angular sampling. EM map colored by local resolution estimates (*left*), Fourier Shell Correlation (FSC) resolution curves (*middle*) and angular distribution plot (*right*) of four cryo-EM reconstructions.

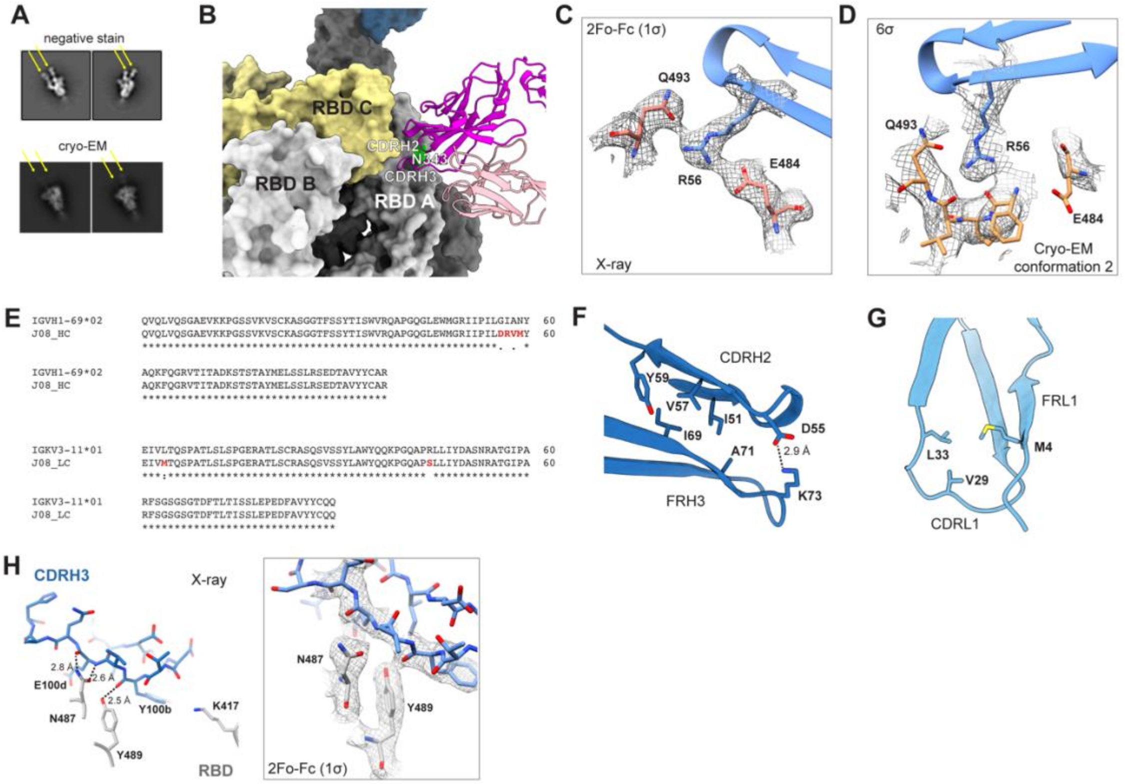

Fig. S4. Key J08 mutations relative to the predicted germline sequence. (**A**) 2D class averages of J08 Fab in complex with SARS-CoV-2-6P-mut7 from negative stain (*top*) and cryo-EM (*bottom*) showing a limited number of Spike trimers with 2 Fabs bound (yellow arrows). (**B**) Alignment of the RBD-J08 crystal structure onto RBD C (down) of the cryo-EM conformation 3 reveals a clash with neighboring RBD-down involving CDRH2 and CDRH3. (**C**) Electron density map highlighting CDRH2 R56 interactions in the J08-RBD x-ray structure. (**D**) Cryo-EM map of CDRH2 R56 and surrounding RBD residues from cryo-EM conformation 2. (**E**) Sequence alignment of J08 heavy and light chains with their respective predicted germline V genes reveals 4 mutations in CDRH2, and 2 mutations in the framework regions of the light chain. (**F**) The heavy chain G55D and A57V mutations stabilize the interaction between CDRH2 and FRH3. (**G**) An L4M mutation in the J08 light chain might stabilize CDRL1 through additional hydrophobic interactions. (**H**) Interactions between RBD and J08 CDRH3 in the x-ray structure. Predicted hydrogen bonds represented as dotted lines with distances labeled. Electron density map of this region shown in gray box.

A

Origin	UK	South Africa	Brazil	California	California	New York	New York	India	India	South Africa	
Pango Lineage	B1.1.7	B.1.351	P.1	B.1.427	B.1.429	B.1.525	B.1.526	B.1.617	B.1.617.2	B.1.1.529	
WHO Label	Alpha	Beta	Gamma	Epsilon	Epsilon	Eta	Iota	Delta	Delta	Omicron	
NTD	69-70 del<br>Y144 del	D80A<br>D215G	L18F<br>T20N<br>P26S<br>D138Y<br>R190S		S13I<br>W152C	Q52R<br>69-70 del<br>Y144 del	L5F<br>T95I<br>D253G	G142D<br>E154K	T19R<br>G142D<br>156 del<br>157 del<br>R158G	A67V<br>69-70 del<br>T95I<br>142-144 del<br>Y145D	211 del<br>ins214EPE
RBD	E484K<br>N501Y	K417N<br>E484K<br>N501Y	K417T<br>E484K<br>N501Y	L452R	L452R	E484K	S477N<br>E484K	E484Q<br>L452R	L452R	G339D<br>S371L<br>S373P<br>S375F<br>K417N<br>N440K<br>G446S<br>S477N	T478K<br>E484A<br>Q493R<br>G496S<br>Q498R<br>N501Y<br>Y505H
S1+S2*	A570D<br>D614G<br>P681H<br>T716I<br>S982A<br>D1118H	D614G<br>A701V	D614G<br>H655Y<br>T1027I	D614G	D614G	D614G<br>Q677H<br>F888L	D614G<br>A701V	D614G<br>P681R<br>Q1071H	T478K<br>D614G<br>P681R<br>D950N	T547K<br>D614G<br>H655Y<br>N679K<br>P681H<br>N764K	D796Y<br>N856K<br>Q954H<br>N969K<br>L981F

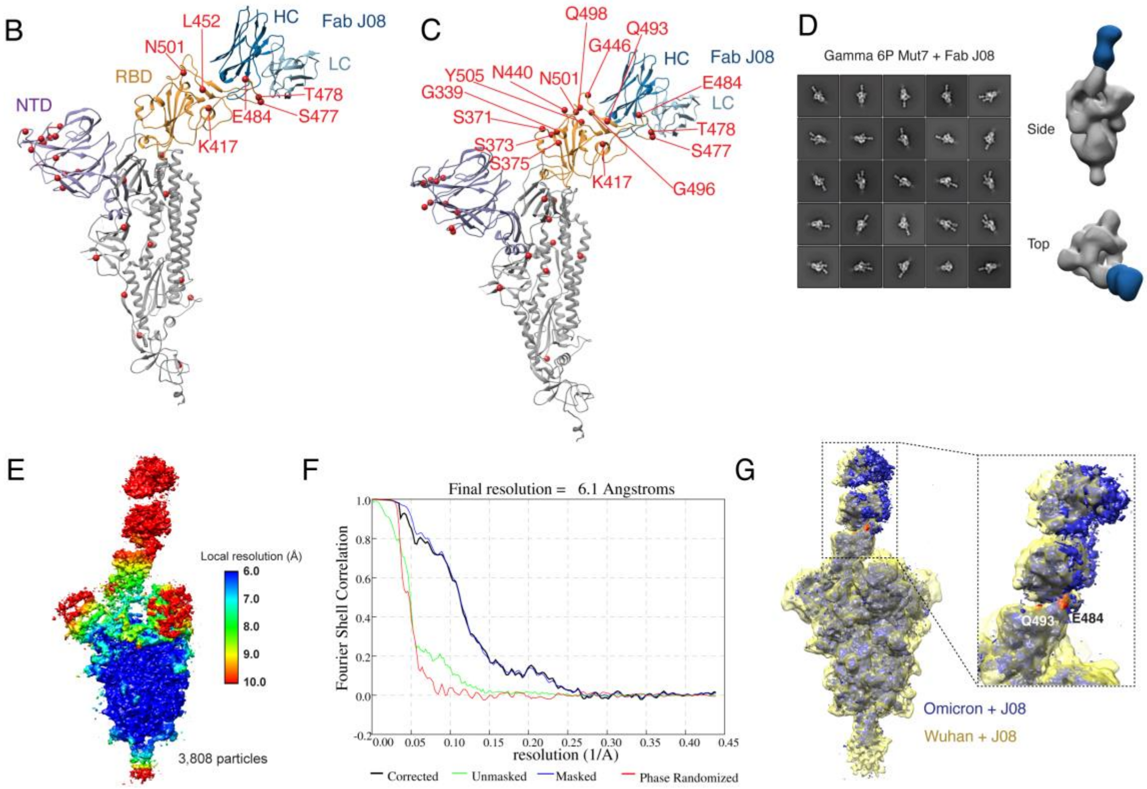

Fig. S5. **J08 binds away from most VOC mutations.** (**A**) Summary table of VOC mutations. NTD and RBD mutations are colored in purple and orange, respectively. Mutations not residing in the NTD or RBD are labelled as S1+S2* and colored in gray. (**B**) Protomer of SARS-CoV-2-Mut2 + Fab J08 with Alpha, Beta, Gamma, Delta, Epsilon, Eta, and Iota mutations represented as red spheres. (**C**) Protomer of SARS-CoV-2-Mut2 + Fab J08 with Omicron mutations represented as red spheres. (**D**) nsEM 2D classes and 3D reconstruction of Gamma 6P Mut7 in complex with Fab J08. J08 Fab density segmented and colored blue. (**E**) Cryo-EM reconstruction of Omicron-CoV-2-6P in complex with J08 Fab, colored by local resolution estimates. (**F**) Fourier shell correlation of Omicron-CoV-2-6P + J08 cryo-EM dataset. (**G**) Comparison of J08-bound SARS-CoV-2-6P-Mut7 and Omicron-CoV-2-6P reconstructions. SARS-CoV-2-6P-Mut7 + J08 map is low pass filtered

to 6 Å and colored yellow. Location of key residues E484 and Q493 shown as red spheres for orientation.

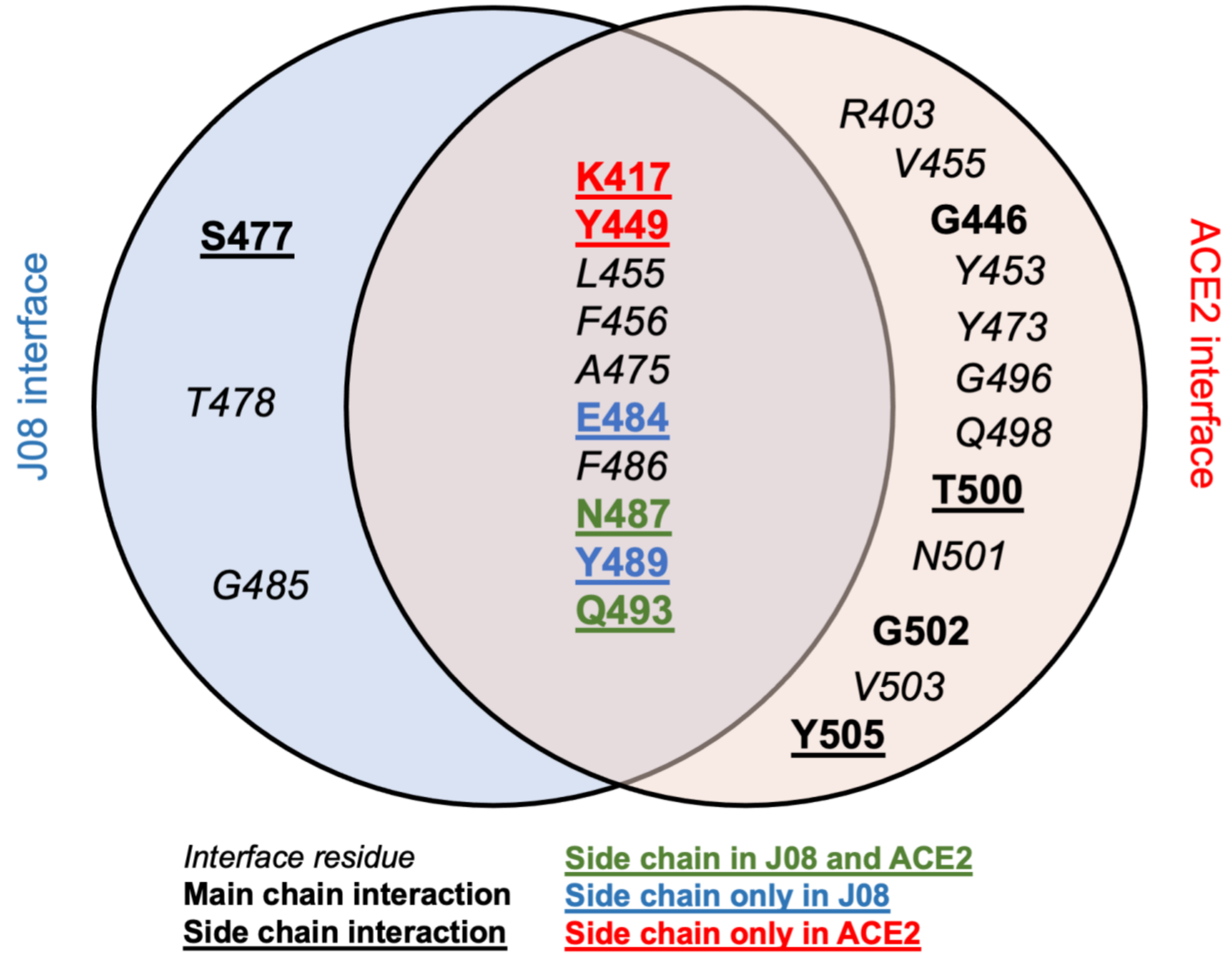

Fig. S6. **Venn diagram depicting shared RBD interface contacts between J08 and ACE2**. Font style and color represents whether molecular interactions between ACE2 or J08 and RBD involve side chain or backbone atoms.

Table S1. Cryo-EM data collection, refinement and model building statistics

**Map**	**SARS-CoV-2-6P-Mut2 S**	**J08 Fab + SARS-CoV-2-6P-Mut2 S (Conformation 1)**	**J08 Fab + SARS-CoV-2-6P-Mut2 S (Conformation 2)**	**J08 Fab + SARS-CoV-2-6P-Mut7 S (Conformation 3)**	**J08 Fab + Omicron-CoV-2-6P**
EMDB	**EMD-24876**	**EMD-24877**	**EMD-24878**	**EMD-24879**	**EMD-26389**
**Data collection**					
Microscope	FEI Talos Arctica			FEI Talos Arctica	Thermo Fisher Glacios
Voltage (kV)	200			200	200
Detector	Gatan K2 Summit			Gatan K2 Summit	Thermo Fisher Falcon 4
Recording mode	Counting			Counting	Counting
Nominal magnification	36,000			36,000	240,000
Movie micrograph pixel size (Å)	1.15			1.15	0.57
Dose rate (e^-/[(camera pixel)*s])	5.5			5.2	4.4
Number of frames per movie micrograph	48			50	40
Frame exposure time (ms)	250			250	150
Movie micrograph exposure time (s)	12.0			12.5	6.0
Total dose (e^-/Å^2)	50			50	49
Defocus range (µm)	-0.2 to -2.4			-0.5 to -2.0	-0.7 to -1.4
**EM data processing**					
Number of movie micrographs	2,325	2,325	2,325	4,090	4,146
Number of molecular projection images in map	27,831	32,769	52,678	43,511	3,808
Box size (pixels)					384
Map voxel size (Å/pixel)	1.15	1.15	1.15	1.15	1.14
Symmetry	C3	C3	C3	C1	C1
Map resolution (FSC 0.143; Å)	3.2	3.4	3.4	4.0	6.0
Map sharpening B-factor (Å^2)	-82.1	-84.3	-91.6	-79.3	0.0
**Structure building and validation**					
Number of atoms in deposited model					
SARS-CoV-2 S protein	25,848	25,176	25,116	22,633	*n/a*
Glycans	924	462	630	420	*n/a*
J08 Fv	N/A	5,157	5,157	1,702	*n/a*
MolProbity score	0.82	0.98	0.95	0.73	*n/a*
Clash score	0.34	0.61	0.74	0.55	*n/a*
Map correlation coefficient	0.85	0.82	0.82	0.73	*n/a*
EMRinger score	4.14	2.74	3.18	1.89	*n/a*
RMSD from ideal					
Bond length (Å)	0.02	0.02	0.02	0.02	*n/a*
Bond angles (˚)	1.72	1.80	1.79	1.73	*n/a*
Ramachandran plot					
Favored (%)	96.80	95.95	96.64	97.83	*n/a*
Allowed (%)	3.20	4.05	3.36	2.17	*n/a*
Outliers (%)	0.00	0.00	0.00	0.00	*n/a*

Side chain rotamer outliers (%)	0.10	0.18	0.00	0.04	*n/a*
PDB	**7s6i**	**7s6j**	**7s6k**	**7s6l**	*n/a*

n/a: not applicable

Table S2. Crystallographic data collection and refinement statistics

**Data collection**	
Beamline	SSRL 12-1
Wavelength (Å)	0.97946
Space group	$P\ 2_1\ 2_1\ 2_1$
Unit cell parameters	
a, b, c (Å)	55.5, 103.2, 123.0
α, β, γ (°)	90, 90, 90
Resolution (Å) [a]	50.0–2.54 (2.58–2.53)
Unique reflections [a]	23,706 (1,144)
Redundancy [a]	7.9 (7.7)
Completeness (%) [a]	99.7 (97.4)
$\langle I/\sigma_I \rangle$ [a]	14.5 (3.7)
R_{sym}[b] (%) [a]	12.8 (50.1)
R_{pim}[b] (%) [a]	4.9 (18.6)
$CC_{1/2}$[c] (%) [a]	98.5 (89.9)
**Refinement statistics**	
Resolution (Å)	40.8–2.53
Reflections (work)	22,417
Reflections (test)	1,166
R_{cryst}[d] / R_{free}[e] (%)	22.0/26.1
No. of atoms	4,904
Macromolecules	4,750
Glycans	14
Solvent	140
Average *B*-value (Å^2)	37
Macromolecules	37
Fab	35
RBD	41
Glycans	59
Solvent	34
Wilson *B*-value (Å^2)	36
**RMSD from ideal geometry**	
Bond length (Å)	0.002
Bond angle (º)	0.454
**Ramachandran statistics (%)**[f]	
Favored	97.4
Outliers	0.0
**PDB code**	7sbu

[a] Numbers in parentheses refer to the highest resolution shell.
[b] $R_{sym} = \Sigma_{hkl} \Sigma_i | I_{hkl,i} - \langle I_{hkl} \rangle | / \Sigma_{hkl} \Sigma_i I_{hkl,i}$ and $R_{pim} = \Sigma_{hkl} (1/(n-1))^{1/2} \Sigma_i | I_{hkl,i} - \langle I_{hkl} \rangle | / \Sigma_{hkl} \Sigma_i I_{hkl,i}$, where $I_{hkl,i}$ is the scaled intensity of the ith measurement of reflection h, k, l, $\langle I_{hkl} \rangle$ is the average intensity for that reflection, and *n* is the redundancy.
[c] $CC_{1/2}$ = Pearson correlation coefficient between two random half datasets.
[d] $R_{cryst} = \Sigma_{hkl} | F_o - F_c | / \Sigma_{hkl} | F_o | \times 100$, where F_o and F_c are the observed and calculated structure factors, respectively.
[e] R_{free} was calculated as for R_{cryst}, but on a test set comprising 5% of the data excluded from refinement.
[f] From MolProbity (*1*).

Table S3. Pairs of J08 and SARS-CoV-2 Spike residues within predicted hydrogen bonding distances. Calculated using PDBePISA (*2*) using a cutoff distance of 3.4 Å.

Conformation 1

#	Antibody region	Ab residue[atom]	RBD residue(atom)	Distance (Å)
1	CDRH2	R50[NH2]	Y489[OH]	3.3
2	CDRH2	I53[O]	Q493[NE2]	3.1
3	CDRH2	R56[NH1]	E484[OE1]	3.4
4	CDRH2	R56[NH2]	F490[O]	3
5	CDRH3	Y100b[OH]	K417[NZ]	3
6	CDRH3	V100c[O]	N487[ND2]	3.4
7	CDRL1	S30[OG]	S477[OG]	3
8	CDRL1	Y32[OH]	N487[OD1]	2.7

Conformation 2

#	Antibody region	Ab residue[atom]	RBD residue(atom)	Distance (Å)
1	FR-H1	G27[N]	T500[O]*	3
2	CDRH1	Y32[OH]	P499[O]*	2.8
3	CDRH2	R50[NH1]	F486[O]	2.9
4	CDRH2	L54[O]	Q493[NE2]	2.9
5	CDRH2	R56[NH1]	F490[O]	2.9
6	CDRH2	R56[[NH2]	Q493[OE1]	2.8
7	CDRH3	A96[O]	N440[ND2]*	3.2
8	CDRH3	E100d[OE2]	Y489[OH]	2.8
9	CDRL1	S30[OG]	S477[OG]	3.3
10	CDRL1	Y32[OH]	S477[N]	3.1

Conformation 3

#	Antibody region	Ab residue[atom]	RBD residue(atom)	Distance (Å)
1	CDRH2	R56[NH1]	F490[O]	3
2	CDRH2	R56[NH2]	L492[O]	3
3	CDRH3	D100[O]	K417[NZ]	3
4	CDRH3	Y100b[O]	Y489[OH]	2.7
5	CDRH3	E100d[N]	N487[OD1]	2.8
6	CDRH3	E100d[O]	N487[ND2]	3.1

X-ray

#	Antibody region	Ab residue[atom]	RBD residue(atom)	Distance (Å)
1	CDRH2	L54[O]	Q493[NE2]	3.1
2	CDRH2	R56[NH1]	E484[OE1]	3
3	CDRH2	R56[NH2]	Q493[OE1]	2.8
4	CDRH3	Y100b[O]	Y489[OH]	2.5
5	CDRH3	E100d[N]	N487[OD1]	2.6
6	CDRH3	E100d[O]	N487[ND2]	2.8

**7**	CDRL1	S30[OG]	S477[O]	3.3
**8**	CDRL1	Y32[OH]	S477[OG]	3

*Interaction involving adjacent protomer

Table S4. List of interface residues between J08 and SARS-CoV-2 Spike. Defined as contributing >5 Å^2 buried surface area as calculated using PDBePISA (*2*).

Heavy chain	**Conformation1**	**Conformation2**	**Conformation3**	**X-ray**
**V2**		RBD*		
**G26**		RBD*		
**G27**		RBD*		
**S31**		RBD*		
**Y32**		RBD*		
**R50**	RBD	RBD	RBD	RBD
**I52**		RBD	RBD	RBD
**I53**	RBD			
**L54**	RBD	RBD	RBD	RBD
**D55**	RBD	RBD	RBD	RBD
**R56**	RBD	RBD	RBD	RBD
**M58**	RBD	RBD	RBD	RBD
**R95**		RBD	RBD	RBD
**A96**		RBD*		
**I97**		RBD*		
**D100**	RBD	RBD*	RBD	RBD
**T100a**	RBD		RBD	RBD
**Y100b**	RBD	RBD	RBD	RBD
**V100c**	RBD	RBD	RBD	RBD
**E100d**	RBD	RBD	RBD	RBD
**Q100e**	RBD	RBD		
**S100f**		RBD	RBD	RBD
**Y102**		RBD*		

Light chain	**Conformation1**	**Conformation2**	**Conformation3**	**X-ray**
**S28**				RBD
**V29**			RBD	RBD
**S30**	RBD	RBD	RBD	RBD
**Y32**	RBD	RBD	RBD	RBD
**T56**		RBD*		
**P91**	RBD	RBD	RBD	RBD
**L92**	RBD	RBD		
**L96**				RBD

RBD	Conformation1	Conformation2	Conformation3	X-ray
**K417**	HC		HC	HC
**N439**		HC#		
**N440**		HC#		
**V445**		HC#		
**Y449**	HC		HC	HC
**L455**	HC	HC	HC	HC
**F456**	HC	HC	HC	HC
**Y473**	HC		HC	
**A475**	HC	HC	HC	HC
**G476**			HC	LC
**S477**	LC	LC	LC	LC
**T478**	LC	LC	LC	LC
**V483**		HC		
**E484**	HC		HC	HC
**G485**	HC	HC	HC	HC
**F486**	HC+LC	HC+LC	HC+LC	HC+LC
**N487**	HC+LC	HC	HC+LC	HC+LC
**Y489**	HC	HC	HC	HC
**F490**	HC	HC	HC	HC
**L492**			HC	
**Q493**	HC	HC	HC	HC
**P499**		HC#		
**T500**		HC#		
**V503**		HC#		
**Q506**		HC#		

*Adjacent protomer RBD
#Contacts involving secondary (adjacent protomer) RBD

SI References

1. C. J. Williams, *et al.*, MolProbity: More and better reference data for improved all-atom structure validation. *Protein Sci.* **27**, 293-315 (2018).
2. E. Krissinel, K. Henrick, Inference of macromolecular assemblies from crystalline state. *J. Mol. Biol.* **372**, 774-797 (2007).
